# Current Strategies of Polyploid Plant Genome Sequence Assembly

**DOI:** 10.3389/fpls.2018.01660

**Published:** 2018-11-21

**Authors:** Maria Kyriakidou, Helen H. Tai, Noelle L. Anglin, David Ellis, Martina V. Strömvik

**Affiliations:** ^1^Department of Plant Science, McGill University, Montreal, QC, Canada; ^2^Fredericton Research and Development Centre, Agriculture and Agri-Food Canada, Fredericton, NB, Canada; ^3^International Potato Center, Lima, Peru

**Keywords:** polyploidy, plant genomics, genome assembly, third generation sequencing, reference genome

## Abstract

Polyploidy or duplication of an entire genome occurs in the majority of angiosperms. The understanding of polyploid genomes is important for the improvement of those crops, which humans rely on for sustenance and basic nutrition. As climate change continues to pose a potential threat to agricultural production, there will increasingly be a demand for plant cultivars that can resist biotic and abiotic stresses and also provide needed and improved nutrition. In the past decade, Next Generation Sequencing (NGS) has fundamentally changed the genomics landscape by providing tools for the exploration of polyploid genomes. Here, we review the challenges of the assembly of polyploid plant genomes, and also present recent advances in genomic resources and functional tools in molecular genetics and breeding. As genomes of diploid and less heterozygous progenitor species are increasingly available, we discuss the lack of complexity of these currently available reference genomes as they relate to polyploid crops. Finally, we review recent approaches of haplotyping by phasing and the impact of third generation technologies on polyploid plant genome assembly.

## Introduction to polyploidy

The fusion of two or more genomes within one nucleus results in polyploidy, resulting in each cell containing more than two pairs of homologous chromosomes. Polyploidy occurs in the majority of angiosperms and is important in agricultural crops that humans depend on for survival. Examples of important polyploid plants used for human food include, *Triticum aestivum* (wheat), *Arachis hypogaea* (peanut), *Avena sativa* (oat), *Musa* sp. (banana), many agricultural *Brassica* species, *Solanum tuberosum* (potato), *Fragaria ananassa* (strawberry), and *Coffea arabica* (coffee). Autopolyploidy results from whole genome duplication, while an allopolyploid is characterized by interspecific or intergeneric hybridizations followed by chromosome doubling (Doyle et al., 2008; Chen, 2010). Genome duplication (autoployploidy) can be a source of genes with novel functions leading to new phenotypes and novel mechanisms for adaptation (Crow and Wagner, 2005). Autopolyploids typically suffer from reduced fertility whereas allopolyploids have potential for heterosis or hybrid vigor (Ramsey and Schemske, 1998). Polyploidy generates great genetic, genomic, and phenotypic novelty (Soltis et al., 2016); however, the higher complexity between genotype and phenotype in polyploids compared to diploid plants makes linking genotype to phenotype a challenging task. For example, allopolyploid plant cells have complex regulatory mechanisms in order to unify gene expression between the homeologs and define their relative contributions to the final phenotype. Hence, polyploidization is one of the major forces of plant evolution and is intimately linked to speciation and diversity (Bento et al., 2011). It is estimated that around 80% of all living plants are polyploids (Meyers and Levin, 2006), while many plant lineages including monocots (i.e., *Oryza*) and eudicots (*Arabidopsis*) have at least one paleo-polyploidy event in their history.

## Overview of the sequencing techniques and their applications in polyploid plant genomes

Genome sequencing was initiated in the mid 1970's with alternative methods to determine the composition of DNA in a target cell or organism (Sanger and Coulson, 1975; Maxam and Gilbert, 1977). The first whole genome to be sequenced was that of a bacteriophage PhiX (Sanger et al., 1977a) with a genome size at 5.3 Kb. However, the revolution in sequencing technology came about when Sanger developed the chain termination or dideoxy method (Sanger et al., 1977b). This technique, now known as Sanger sequencing, was adopted by most molecular biology laboratories and was the primary method of sequencing for 30+ years allowing sequencing of fragments of approximately 800–1,000 bp.

It took over 20 years from the time the first genome of a bacteriophage was sequenced until plant biologists had a draft genome of a flowering plant. First to be sequenced was the genome of *Arabidopsis thaliana*, a small weedy plant (Arabidopsis Genome Initiative, 2000). After the release of the Arabidopsis genome sequence, economically important crops such as *Oryza sativa* (rice), *Carica papaya* (papaya), and *Zea mays* (maize) were sequenced using Sanger sequencing (International Rice Genome Sequencing Project, 2005; Ming et al., 2008; Schnable et al., 2009). Yet, of these plant genomes, only rice and Arabidopsis were sequenced using the Bacterial Artificial Chromosome (BAC) approach, and thus, are more complete genomes, whereas the others are drafts in a less completed stage (Claros et al., 2012).

The diploidized tetraploid genome of *Glycine max* (soybean) was the first polyploid plant genome released (publicly available in early 2008, Schmutz et al., 2010), followed by the tetraploid *Arabidopsis lyrata* (Hu et al., 2011) (Table 1). The soybean project was very costly, and the resulting assembly consisted of the largest published plant genome performed using the Sanger Whole Genome Sequencing (WGS) method. In 2011, the genome of *Jatropha curcas* (an oil-bearing tree) that has variable ploidy levels (Table 1), was also sequenced using the Sanger method (Sato et al., 2010). The assembly of the complex tetraploid genome of cultivated cotton—*Gossypium arboretum* (Li et al., 2014) was followed by the reference genome of wheat, derived from the assembly of the large complex genome of *Aegilops tauschii*, one of the three diploid progenitors of bread wheat (Zimin et al., 2017).

**Table 1:** Sequenced plant polyploid genomes through May 2018.

**NA**	**Organism name**	**Genome size (Mb)**	**Current status**	**1st Release date in NCBI**	**Ploidy level**	**References/center**
1	*Arabidopsis lyrata* subsp *lyrata*	206.823	Scaffold	2009-11-30	Tetraploid	Hu et al., 2011
2	*Glycine max*	978.972	Chromosome	2010-01-05	Allotetraploid	Schmutz et al., 2010
3	*Triticum aestivum*	15344.7	Chromosome 3B	2010-07-15	Allohexaploid	Choulet et al., 2010
4	*Solanum tuberosum*	705.934	Scaffold	2011-05-24	Autotetraploid	Potato Genome Sequencing Consortium, 2011
5	*Actinidia chinensis*	604.217	Contig	2013-09-16	Tetraploid	Huang et al., 2013
6	*Fragaria orientalis*	214.356	Scaffold	2013-11-27	Tetraploid	Hirakawa et al., 2014
7	*Fragaria x ananassa*	697.762	Scaffold	2013-11-27	Allooctaploid	Hirakawa et al., 2014
8	*Beta vulgaris*	566.55	Chromosome	2013-12-18	2n, 4n (Beyaz et al., 2013)	Dohm et al., 2014
9	*Oryza minuta*	45.1659	Chromosome	2014-04-16	Tetraploid	Oryza Chr3 Short Arm Comparative Sequencing Project
10	*Camelina sativa*	641.356	Chromosome	2014-04-17	Hexaploid	Kagale et al., 2014
11	*Brassica napus*	976.191	Chromosome	2014-05-05	Allotetraploid	Chalhoub et al., 2014
12	*Brassica oleracea* var. oleracea	488.954	Chromosome	2014-05-22	Hexaploid	NCBI
13	*Nicotiana tabacum*	3643.47	Scaffold	2014-05-29	Allotetraploid	Sierro et al., 2014
14	*Eragrostis tef*	607.318	Scaffold	2015-04-08	Allotetraploid	Cannarozzi et al., 2014
15	*Gossypium hirsutum*	2189.14	Chromosome	2015-04-29	Allotetraploid	Li F. et al., 2015
16	*Zoysia japonica*	334.384	Scaffold	2016-03-15	Tetraploid	Tanaka et al., 2016
17	*Zoysia matrella*	563.439	Scaffold	2016-03-15	Allotetraploid	Tanaka et al., 2016
18	*Zoysia pacifica*	397.01	Scaffold	2016-03-15	Allotetraploid	Tanaka et al., 2016
19	*Musa itinerans*	455.349	Scaffold	2016-05-21	2n, 3n hybrids (Wu et al., 2016)	South China Botanic Garden, CAS
20	*Rosa x damascena*	711.72	Scaffold	2016-06-13	Tetraploid	BIO-FD & C CO., LTD
21	*Chenopodium quinoa*	1333.55	Scaffold	2016-07-11	Tetraploid	Jarvis et al., 2017
22	*Brassica juncea* var. tumida	954.861	Chromosome	2016-07-19	Allotetraploid	Zhejiang University
23	*Hibiscus syriacus*	1748.25	Scaffold	2016-07-29	2n, 3n, 4n (Van Huylenbroeck et al., 2000)	Korea Research Institute of Science and Biotechnology (Kim et al., 2017)
24	*Gossypium barbadense*	2566.74	Scaffold	2016-10-28	Tetraploid	Huazhong Agricultural University
25	*Momordica charantia*	285.614	Scaffold	2016-12-27	2n to 6n (Kausar et al., 2015)	Urasaki et al., 2016
26	*Drosera capensis*	263.788	Scaffold	2016-12-30	Tetraploid (Rothfels and Heimburger, 1968)	Butts et al., 2016
27	*Capsella bursa-pastoris*	268.431	Scaffold	2017-01-29	Tetraploid	Lomonosov Moscow State University
28	*Saccharum* hybrid cultivar	1169.95	Contig	2017-03-03	It varies (D'Hont, 2005)	Riaño-Pachón and Mattiello, 2017
29	*Xerophyta viscosa*	295.462	Scaffold	2017-03-31	Hexaploid	Costa et al., 2017
30	*Triticum dicoccoides*	10495	Chromosome	2017-05-18	Tetraploid	WEWseq consortium
31	*Utricularia gibba*	100.689	Chromosome	2017-05-31	16-ploid	Lan et al., 2017
32	*Eleusine coracana*	1195.99	Scaffold	2017-06-08	Allotetraploid	Hittalmani et al., 2017
33	*Dioscorea rotundata*	456.675	Chromosome	2017-07-28	Tetraploid	Iwate Biotechnology Research Center
34	*Ipomoea batatas*	837.013	Contig	2017-08-26	Autohexaploid	Yang et al., 2017
35	*Echinochloa crus-galli*	1486.61	Scaffold	2017-10-23	Hexaploid	Zhejiang University
36	*Pachycereus pringlei*	629.656	Scaffold	2017-10-31	Autotetraploid	Zhou et al., 2017
37	*Olea europaea*	1141.15	Chromosome	2017-11-01	2n, 4n, 6n (Besnard et al., 2007)	Unver et al., 2017
38	*Monotropa hypopitys*	2197.49	Contig	2018-01-03	Hexaploid	Institute of Bioengineering, RAS
39	*Dactylis glomerata*	839.915	Scaffold	2018-01-19	Autotetraploid	Sichuan Agricultural University
40	*Panicum miliaceum*	848.309	Scaffold	2018-01-23	Allotetraploid	China Agricultural University
41	*Euphorbia esula*	1124.89	Scaffold	2018-02-06	Hexaploid	USDA-ARS
42	*Santalum album*	220.961	Scaffold	2018-02-12	2n, 4n etc (Xin-Hua et al., 2010)	Center for Cellular and Molecular Platforms
43	*Avena sativa*	67.3266	Contig	2018-02-26	Hexaploid	The Sainsbury Laboratory
44	*Panicum miliaceum*	850.677	Chromosome	2018-04-09	Tetraploid	Shanghai Center for Plant Stress Biology
45	*Arachis monticola*	2618.65	Chromosome	2018-04-23	Tetraploid	Henan Agricultural University
46	*Arachis hypogaea*	2538.28	Chromosome	2018-05-02	Allotetraploid	International Peanut Genome Initiative
47	*Artemisia annua*	1792.86	Scaffold	2018-05-08	Tetraploid	Shen et al., 2018

*The release date refers to the first release of the genomes in NCBI, before any improvement of the assemblies. Some have been updated after this date*.

Next Generation Sequencing (NGS) technologies became commercially available in 2004 (Mardis, 2008) reducing sequencing costs and increasing massively sequencing throughputs, but also expanding the complexity of fragment assembly due to its short-sequence read output. NGS allows genome sequencing to be performed with lower DNA concentrations and thus, has applications in genome sequencing and re-sequencing, metagenomics, transcriptomics (RNA-sequencing) and even in personal genomics (personal medicine). These techniques can reduce the gap between genotype and phenotype by combining for example genomics and transcriptomics data. Some of the NGS platforms that have been employed in recent years include: 454 or pyro-sequencing (by Roche, Basel, Switzerland, with read lengths up to 700 bp), SOLiD (by Life Technologies, Carlsbad, California, 50 bp), HiSeq (by Illumina, San Diego, California, 2 × 250 bp), MiSeq (by Illumina, 2 × 300 bp) and Ion Torrent/Proton (by Life Technologies, 200 bp). NGS technologies are advantageous because, unlike Sanger sequencing, DNA cloning is not required making the process simpler, with greater adaption for a broad range of biological phenomena, and massive parallelization at decreased costs. However, NGS does suffer from some disadvantages: the short sequence length requires unique assembly algorithms, base calling is less accurate than Sanger sequencing, and the quality of NGS assemblies is lower than those made from Sanger sequence (Claros et al., 2012). Examples of polyploid plant genomes sequenced using Illumina technology are the first assembly of the hexaploid *T. aestivum* (wheat) genome (Choulet et al., 2010), and the genome of *G. hirsutum* (cotton) (Li et al., 2015). The genomes of *Brassica oleracea* (cabbage) and *B. napus* (rapeseed) (Chalhoub et al., 2014) were sequenced with a combination of 454 and Illumina technologies. A genome assembly service using only high-quality short Illumina reads is offered by NRGene's DenovoMAGIC platform (http://www.nrgene.com/technology/denovomagic/). The recently annotated allohexaploid wheat genome was constructed using DenovoMAGIC2 (International Wheat Genome Sequencing Consortium (IWGSC), 2018). The latest version; DenovoMAGIC v 3.0 promises production of long, phased scaffolds using only NGS.

The emergence of the Third Generation Sequencing technologies consists of the most recent genome sequencing approaches, characterized by long reads. These methods have further reduced sequencing costs, simplified preparatory and sequencing methods (Schadt et al., 2010), while providing longer read lengths, typically measured in kilo bases (Kb) rather than bases (bp). While there are many upsides to this new technology, caveats include high error rates and a requirement for very high-quality DNA. However, these approaches currently look promising in meeting the challenges of sequencing and assembling large, repetitive, and complex plant genomes by the production of large quantities of long reads to help bridge difficult regions in the genome. There are currently two types of technologies included in the Third-Generation sequencing approaches: long-read sequencing and long-range scaffolding technologies (Jiao and Schneeberger, 2017).

Among the long-read sequencing technologies, the most widely used technology is the Pacific Biosciences' Single Molecule Real-Time (SMRT), with an average read length 20 Kb. For the assembly of the *Chenopodium quinoa* genome, a read length of ~12 Kb was reported using this technology (Jarvis et al., 2017). Additionally, Illumina introduced another long-read technology, the Synthetic Long-Reads (SLR) from short-read sequencing data, with a median length of 8–10Kb (Table 2). However, a maximum length of ~21 Kb was achieved in a sugarcane hybrid sequencing project (Riaño-Pachón and Mattiello, 2017). SLR can be used to resolve the haplotype of individuals, which is highly desired in the case of polyploid plant genomes. Finally, Nanopore, introduced by Oxford Nanopore Technologies, can generate a median length greater than 5 Kb, however a ~12 Kb median length was reported while sequencing the wild *Solanum pennellii* genome (Schmidt et al., 2017).

**Table 2:** Third generation sequencing platforms.

**Technology**	**Reads**	**Drawbacks**	**Plant assembly**
PacBio	Single molecule long-reads, average length ~ 10–18 Kb	False insertions in the raw reads, high error rate. Error correction algorithms are required	*Chenopodium quinoa* (Jarvis et al., 2017)
Oxford Nanopore	Single molecule long-reads, average length ~ 10 Kb, max 100 Kb	Raw reads with false deletions and homopolymer errors. Requirement for error correction algorithms	*S. pennellii, A. thaliana, O. coaectata* (Mondal et al., 2017; Schmidt et al., 2017; Michael et al., 2018)
Illumina Synthetic Long reads	Synthetic long-reads derived from the short sequencing reads, average length ~ 100 Kb	High rate false indels (insertions, deletions). They require good trimming, correction algorithms	*Saccharum* sp. (Riaño-Pachón and Mattiello, 2017)
10X Genomics	Linked reads derived from short-read sequences, average length ~ 100 Kb^*^	Needs designed algorithms and aligners, poor resolution of locally repetitive sequences. Sparse sequencing	*Capsicum annuum* (Hulse-Kemp et al., 2018)
BioNano Genomics	Optical mapping of long, fluorescently labeled DNA fragments, average length ~ 250 Kb	Not many algorithms available for a reliable alignment between the optical map and the genome assembly	*Brassica juncea* (Yang et al., 2016)
Hi-C	Pairs short reads with an average length ~ 100 bp, method originally developed to study the 3D folding of the genome	Scattered sequencing with variable genomic distance between pairs	*Triticum aestivum* (International Wheat Genome Sequencing Consortium (IWGSC), 2014)

* *10X Genomics is very similar to Illumina's SLR, with the difference that 10X Genomics can process more and larger fragments and the assemble of the different fragments does not necessarily depend on the sequencing coverage. Illumina's SLR system synthesizes the sequences of DNA fragment in contrast to 10x Genomics where the reads show only a part of DNA fragments. NA, not applicable*.

Even with the rapid progress and improvement of long-read technologies, it is still not possible to assemble a complete diploid plant genome using only NGS sequencing reads (Jiao and Schneeberger, 2017). Hence, long-range scaffolding technologies are essential for improving the contiguity of an assembly, which requires the extension of the contigs into scaffolds and eventually their alignment into chromosomes. Based on currently available sequencing technologies, additional genetic and physical maps are required. An alternative approach is based on chromosome conformation capture sequencing (Hi-C) provided by Dovetail Genomics (https://dovetailgenomics.com/) and PhaseGenomics (https://phasegenomics.com/), which creates long-range mate pair data for NGS (Lieberman-Aiden et al., 2009; van Berkum et al., 2010). The generated data can be used for phasing and scaffolding, which captures the entire eukaryotic chromosomes when they are combined with high quality draft assemblies (Sedlazeck et al., 2018). Genome phasing is the identification of the alleles in each of the chromosomes. The most recent announcement of the PhaseGenomics Biotechnology company is its collaboration with Pacific Biosciences for the release of FALCON-Phase (Kronenberg et al., 2018). FALCON-Phase tool promises to solve the haplotyping problem in diploids, by enabling the construction of fully-phased chromosome-scale assemblies by combining SMRT long reads and Hi-C data. The latest technology is from GemCode, introduced by 10X Genomics in 2015 (www.10xgenomics.com). This approach is similar to the SLR protocol of Illumina, but it can process longer fragments and it does not require as much read depth as the SLR. The average read length captured with this approach can be greater than 100 Kb (Table 2).

## Challenges of polyploid genome assembly

A reference genome is a digital, linear nucleic acid sequence containing only a single set of chromosomes plus any unanchored heterozygous contigs and/or scaffolds. A reference genome is used to observe variations across different individuals within a species, to study evolution and to aid genome assembly. In the case of a polyploid genome, things become more complicated. For an allopolyploid organism, a reference genome contains the assembled DNA sequences of the ancestors subgenomes (e.g., *F. ananassa, B. napus, A. hypogaea, G. hirsutum, and T. aestivum*) in addition to any unanchored sequences that are kept in additional pseudochromosome(s) (e.g., *T. aestivum, S. tuberosum*), and for an autopolyploid organism the genome that went through the duplication event(s) (e.g., *S. tuberosum*) in addition to any unanchored sequences. It does not necessarily represent any allelic variation present in the individuals. When high throughput sequencing reads are mapped to a reference genome, alternate alleles can be retrieved from each genomic region, based on the sequencing coverage and diversity in the individual compared to the reference. These alternate alleles for an organism can be detected and used for haplotype assembly for each of the present haplotypes. Polyploid assembly is similar to the sum of a number of problems of haplotype reconstruction (Aguiar and Istrail, 2013); hence, the computational complexity increases with higher ploidy. This means that the genome assembly of an n-ploid organism will result in the construction of n numbers of haplotypes. This is not an easy task as the knowledge of one haplotype does not automatically determine how to phase others (Motazedi et al., 2017).

Whole-genome duplication events have also been associated with genome rearrangement, atypical recombination, transposable element activation, meiotic/mitotic defects, and intron expansions and DNA deletion (Hufton and Panopoulou, 2009). The assembly of autopolyploid genomes is extremely challenging as fragments of a subgenome might be assigned to the wrong subgenome, which results in misassembled false genomes. Allopolyploids may present the same challenge, but given the greater genetic distance, resolving their subgenomes is likely less problematic during assembly. These events multiply the regular challenges of plant genome sequence assembly, such as repeat content, transposable elements, high heterozygosity, gene content and gene families of non-coding RNAs due to their repetitiveness after duplication events and the fact that their detection is crucial for proper genome annotation.

Polyploidization can lead to higher levels of heterozygosity, which can be confounded in asexually propagated plants such as potato causing greater difficulties in the identification of haplotypes. This is due to multiple alleles from the same locus being mistaken as sequences from different loci (Huang et al., 2017). This is especially problematic when using short sequence reads for genotyping or genome assembly, because the results will be highly fragmented assemblies with a total assembly size longer than expected. In addition, contigs can break at polymorphic regions or misassemblies can occur between large-scale duplications (Claros et al., 2012). This assembly problem is not unique to polyploid plants, however and can also occur in plants with segmental genome duplications.

The ploidy level of the plant genome must be carefully considered when choosing the appropriate assembly algorithm. The presence of two or more sets of genes within the same nucleus can affect the accuracy of the assembly, making it difficult to differentiate between homologs or homeologs (Claros et al., 2012). Glover et al. (2016) define homeologs as pairs of genes or chromosomes in the same species, derived by speciation but brought back to the same genome after a polyploidization event(s). Identifying functionally conserved homeologs however, provides important genetic material for crop improvement in many crops, including *Musa acuminata* (banana), *S. tuberosum* (potato), *Gossypium hirsutum* (cotton) and *T. aestivum* (wheat) (Chen and Dubcovsky, 2012; Glover et al., 2016). Examples of how polyploids also confer emergent properties are seed oil accumulation in *Brassica napus* (canola), spinnable fibers in cotton, and grain composition in wheat (Michael and VanBuren, 2015).

As mentioned above, several complex polyploidy plant genomes have been sequenced. The decreasing costs of NGS technologies led to the sequencing and assembly of a number of polyploid plant genomes using these technologies (Table 1). Based on NCBI database (data retrieved on the 4th of July 2018: https://www.ncbi.nlm.nih.gov/genome/browse/#!/overview/), 320 land plants, 47 of which are polyploid, have been sequenced (as of 4th of July of 2018). Of the 72 assembled in 2017, 19 are polyploid, and three were released in January 2018. Only 16 polyploid plant genomes have been assembled into chromosomes, 26 assembled into scaffolds, and the rest (5) are still contigs (Table 1).

## Technology-related challenges

**Figure 1:**
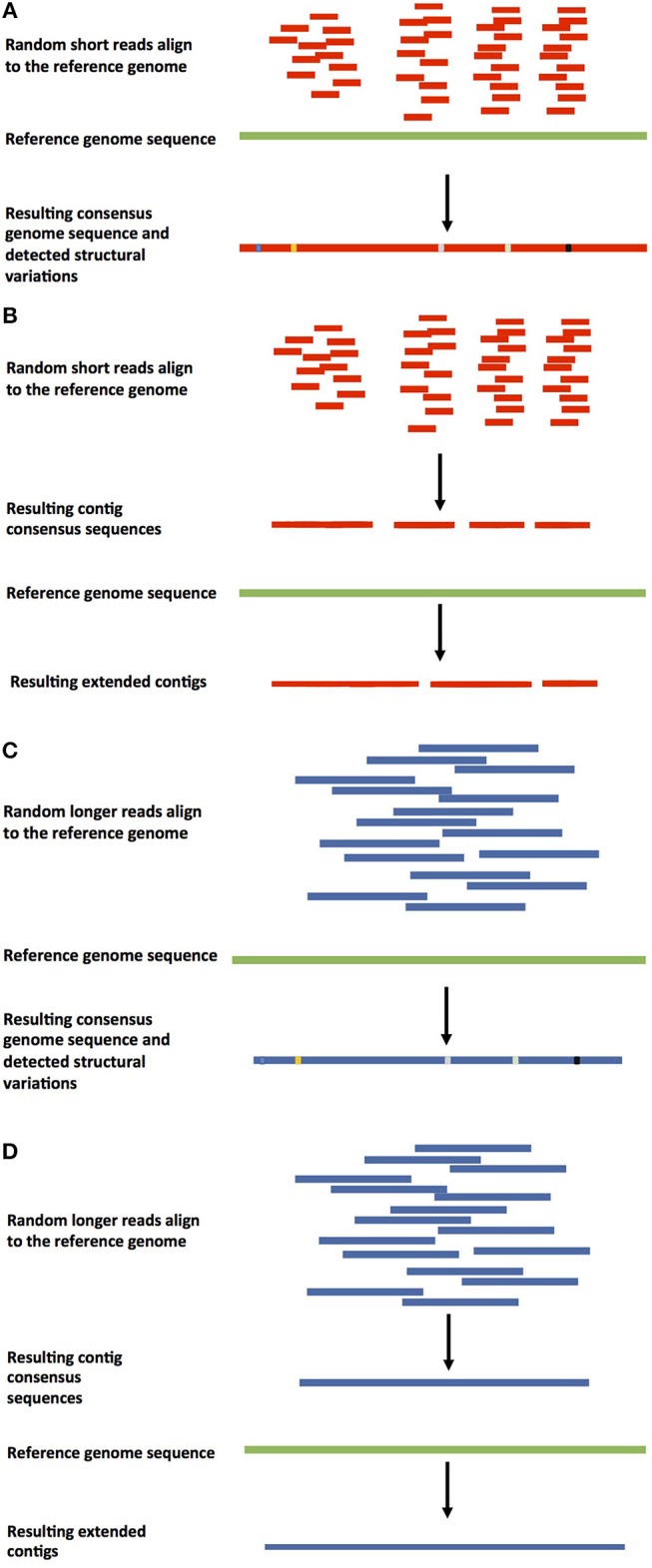
Approaches for reference-based genome assembly. **(A)** Shorter-read guided assembly. In this method, shorter reads are aligned against the reference genome, a consensus assembly is generated, and structural variations are detected. It can also be used to detect contamination in the sequenced reads. This approach is used when genomes are re-sequenced to detect polymorphisms in individuals. **(B)** Guided *de novo* genome assembly of shorter reads. Previously *de novo* assembled shorter reads are aligned against the reference or a closely related genome to extend the existing contigs. **(C)** Longer-read guided assembly. Longer reads are aligned against the reference genome, a consensus genome assembly is constructed, and structural variations are detected. **(D)** Guided *de novo* genome assembly of longer reads. Longer reads are *de novo* assembled into contigs, which are aligned against the reference or a closely related genome to be extended.

**Figure 2:**
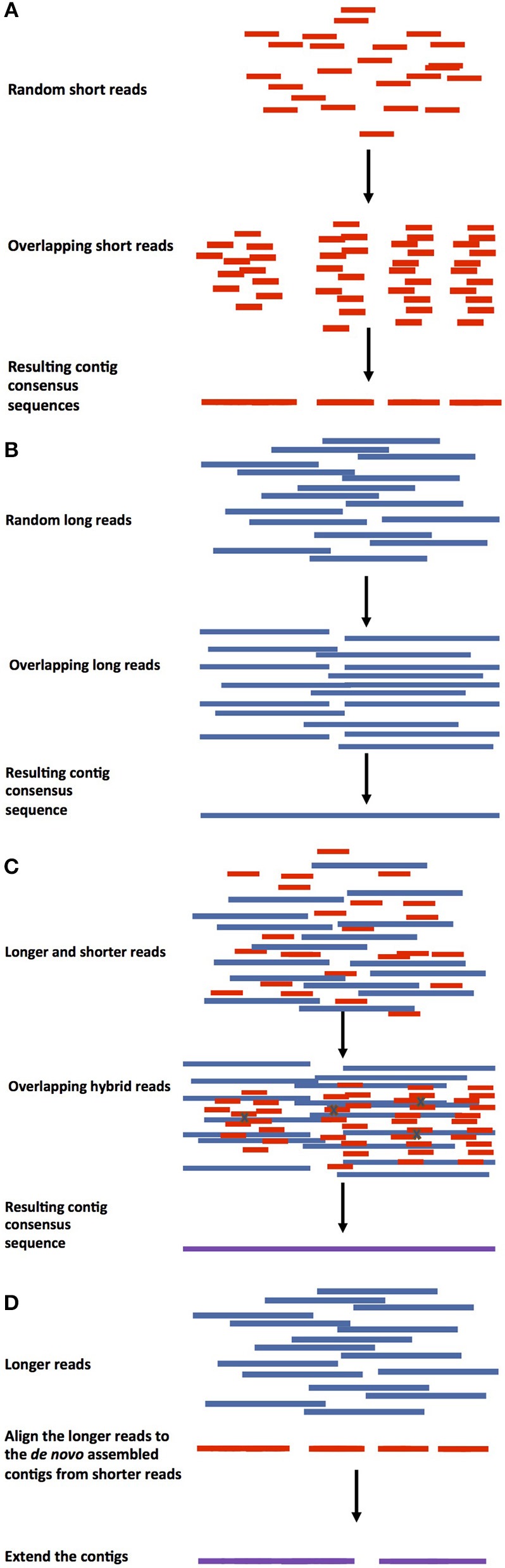
Approaches for *de novo* assembly genome approaches. **(A)** Short read assembly. Genome assembly using only shorter reads and any assembly tool to construct contiguous sequences/contigs. **(B)** Longer reads assembly. Contig (red) assembly using longer reads (long, linked reads, optical maps) followed by scaffold assembly and gap filling. **(C)** Hybrid genome assembly. In this method, shorter reads can be assembled into contigs and the longer reads can be used for error correction (errors represented by Xs), then the corrected contigs can be assembled into scaffolds and the gaps filled. **(D)** Hybrid genome assembly using pre-assembled contigs. Longer reads are aligned against *de novo* pre-assembled contigs from shorter reads, followed by contig extension.

There are two basic approaches to genome assembly. Comparative assembly is a reference guided method that uses the sequences of already assembled related organisms, a reference genome, for guidance. *De novo* assembly targets organisms that have not been sequenced before (Pop, 2009), putting together the pieces without guidance from a prior reference genome. The two approaches are not completely mutually exclusive, because even in cases where reference genomes are available, regions that varied in the newly sequenced target genome need to be assembled *de novo*. Different approaches of guided and *de* novo genome assemblies can be found in Figures 1, 2. The reference guided comparative assembly approach (Figure 1) can be performed in two ways: mapping short or long reads against the reference to construct a consensus (Figures 1A,C) or assembling the reads *de novo* and then use the reference genome to orientate the resulting contigs or scaffolds in an alignment and identify misassembled regions (Figures 1B,D) (Lischer and Shimizu, 2017).

The reference-based comparative assembly approach is usually used when genomes are re-sequenced, or to correct misassemblies or extend existing contigs of already assembled genomes (Figures 1B,D), and also for variant detection (Figures 1A,C) and haplotype construction. An assembled genome sequence is used as a reference and the sequenced reads are independently aligned against this sequence. Dynamic programming is used to identify the optimal alignment for the candidate positions that match the best. Structural variations (such as insertions or deletions) in the re-sequenced genome(s) tend to increase the complexity of the alignment. The resulting alignment allows the extraction of the structural variants and construction of the haplotypes.

The *de novo* genome assembly method is applied when a reference genome sequence does not exist for a closely related species. In this case, the genome sequence is constructed through overlapping sequenced reads, usually using graph-based algorithms. It is difficult to perform *de novo* genome assembly, especially when only shorter reads are available. Both single end (SE) and paired-end (PE) reads are difficult to assemble *de novo*, with SE reads being slightly more challenging (i.e., Illumina, Figure 2A). Long range reads can be used (Figure 2B), or a hybrid approach can be applied, where shorter and longer reads can be used together for a better assembly (Figures 2C,D). As for the assessment, there are currently no unified assembly quality metrics to assess the quality of the *de novo* generated assembly, although one value that is commonly used is the N50. The value of N50 is a weighted median for when at least 50% of the assembly is contained in contigs or scaffolds of equal or greater length.

In general, the comparative method requires less computation as the sequenced reads are aligned to a reference genome. However, significant bias can occur in the comparative genome approach, as divergent (duplicated) regions of the genome may not get reconstructed properly, and thus, may completely miss the diversity present in the newly assembled genome (Lischer and Shimizu, 2017). In contrast, the *de novo* genome assembly even for a diploid genome is classified as an “NP-hard” (non-deterministic polynomial-time) problem meaning it does not have an optimal, known solution. The genome assemblers must assemble a jigsaw puzzle of very small pieces. These pieces are the short reads (~75–300 bp) and different assembly tools are used to resolve a best-fit assembly. However, given that it is a NP-hard problem, most assemblies are likely only an approximation of the true genome order.

The assemblers also face the challenge of the repetitive nature of plant genomes along with heterozygosity and haplotype ambiguity that frequently splits these regions into multiple contigs. A number of algorithms are used for this computation. Some of the most well-known are the overlap computation, the Greedy algorithm (Huson et al., 2002), the Eulerian path (Pevzner et al., 2001), and two classes of assembly algorithms: Overlap-Layout-Consensus (OLC) and de Brujin graph. The overlap computation within an assembly tool requires a great deal of computational time, which can be easily reduced by parallelizing the computations using multi-processor machines or servers (Pop, 2009). The complexity of the overlap computation is affected by the number of the input sequencing reads. Furthermore, the assemblers based on the Greedy algorithm give the simplest (Pop, 2009), most intuitive solution to the assembly problem, yet it is harder to prove the correctness of the algorithm even if the algorithm is correct (Pop, 2009).

The OLC, which can effectively assemble very short reads, has been one of the most successful assembly strategies. The Eulerian approach was proposed as an alternative to the OLC for the assembly of Sanger data; however, because of its sensitivity to sequencing errors it has not been extensively used (Pop, 2009). Overall, the short sequence reads need to be assembled into contigs, then the contigs need to be placed into bigger scaffolds, and finally chromosomes. Examples of tools that use the OLC algorithm in combination with other techniques is MASURCA that uses de Brujin graphs to construct mega-reads for a better assembly (Zimin et al., 2017) and BAUM that uses adaptive unique mapping to reconstruct repetitive regions (Wang et al., 2018).

*De novo* genome assembly is essential to capture the biological diversity within re-sequenced genomes. Yet, this task is near impossible without the use of mate-pairs, longer reads, or linked reads to provide information that can bridge these difficult repetitive regions. Currently, there is a lack of genome assembly and mapping algorithms specialized for polyploid genomes. These would need to be optimized for using more computational power (resources) to handle the challenge of the increased complexity and size of the data sets. Polyploid genome assemblies made from only short reads fail to capture haplotyping variation and present only a single consensus sequence of several chromosome sets. Better algorithms are necessary to minimize misassembly of paralogous and orthologous regions in polyploid plant genomes.

Sequencing errors, read length, quality values, number of reads, and coverage are important factors in assembling genomes and there is little difference in these factors/variables between diploid and polyploid plant genomes. However, because of the complex nature of polyploid genomes, there is not “a best fit” for the main assembling pipeline and not every approach is reproducible for other polyploid plant genomes. Different results can be obtained from the various algorithms used for alignment and assemblers and often genome assemblies are only an estimate of the true biological genome. It often takes a decade or longer to make improvements and corrections to the original draft release. For example, the human genome released in 2000 has gone through multiple revisions to correct errors. Furthermore, the metrics used to make comparisons tend to only focus on size which does not capture contig quality nor accuracy, and thus, there are no commonly accepted standardized methods for validation of the assemblers, which means most genomes are accepted as “draft” assemblies (Narzisi and Mishra, 2011). BUSCO (Simão et al., 2015) and QUAST (Gurevich et al., 2013) are two examples of tools that have been created in an attempt to validate the quality of an assembly.

## How to estimate ploidy level in plants

The ploidy level in plants is normally estimated by measuring the *C*-value (amount of DNA in the unreplicated gametic nucleus) using flow cytometry (Dart et al., 2004; Eaton et al., 2004; Grundt et al., 2005; Clarindo et al., 2008; Harbaugh, 2008; reviewed by Yang et al., 2011). For example, flow cytometry was used to estimate genome content and ploidy in over 300 accessions of the Magnoliaceae family (Parris et al., 2010), in six *Olea europea* (olive) subspecies (Besnard et al., 2007), and in *B. napus* leaf tissue samples (Cousin et al., 2009). Public databases exist to capture *C*-value and ploidy levels in plants (e.g., http://data.kew.org/cvalues/). Recent tools have also been developed to infer the ploidy level using NGS data, such as ploidyNGS (Dos Santos et al., 2017), ConPADE (Margarido and Heckerman, 2015), and a pipeline using single nucleotide polymorphism (SNP) counts that was reported earlier by Yoshida et al. (2013) for the estimation of ploidy level in the plant pathogen *Phytophthora infestans*. A general approach to estimate ploidy levels using NGS is by mapping the sequenced reads to the reference genome and then counting the number of mapped reads, representing the different alleles at each position. PloidyNGS (Dos Santos et al., 2017) was implemented by automating the process of observing the frequency of the alleles by generating a histogram. It was tested on diploid and haploid *Saccharomyces cerevisiae* datasets. ConPADE (Margarido and Heckerman, 2015) was specifically designed to estimate the ploidy levels of highly polyploid plant genomes and has been tested on wheat. A weakness is its sensitivity to the quality of the mapping step as this can bias the ploidy estimation (Dos Santos et al., 2017). Finally, the pipeline by Yoshida et al. (2013) is similar in the sense that the distribution of read counts at biallelic SNPs is observed, which allowed the identification of diploid, triploid, and tetraploid *P. infestans* strains. Another recent statistical tool for ploidy estimation is nQuire (Weiß et al., 2017), which uses NGS data to distinguish between diploids, triploids and tetraploids.

Ploidy estimation tools have been reported such as EAGLE (Loh et al., 2016) and ReadSim (Schmid et al., 2006). More recent tools for the haploid assembly consist of HapCompass (Aguiar and Istrail, 2012), HaploSim (Bastiaansen et al., 2012), HapCut (Bansal and Bafna, 2008), and HapCUT2 (Edge et al., 2017). Real and simulated data were analyzed with HapCUT2 (Edge et al., 2017) and it was shown that it is more accurate and can use not only WGS, but also SMRT (www.pacb.com//smrt-science) and Hi-C data (Lieberman-Aiden et al., 2009) for haplotype assembly. SWEEP (Clevenger and Ozias-Akins, 2015) is a tool designed to filter SNPs detected in re-sequenced autopolyploid and allopolyploid crops using NGS approaches. The detected SNPs can be further used for the haplotype construction. Another NGS tool is HANDS (Mithani et al., 2013), which also can be used for auto- and allopolyploids and by aligning the sequenced reads to the reference genome(s) it can detect the subgenomes in polyploids. Longranger software by 10X Genomics can be used for phasing. It can determine which barcodes are associated with each heterozygous locus and while phasing, it can construct the organism's haplotypes. Simply, it aligns the raw reads to the sequence of both alleles to determine which allele each read represents.

## How to “resolve” the ploidy issue (how to reduce the complexity of the problem)

### Genome-related approach

Several strategies have been adopted for the sequencing and assembly of large polyploid genomes of crop plants (Bevan et al., 2017). One approach involves the reduction of genome complexity using a natural or *in vitro* generated haploid. An example is the sequencing of the potato genome by the Potato Genome Sequencing Consortium (2011). This genome was produced from a doubled monoploid that was homozygous for a single set of 12 chromosomes to generate a reference (The Potato Genome Sequencing Consortium, 2011). A similar approach was used for the genome assembly of the hexaploid bread wheat, *T. aestivum*. Aneuploid bread wheat lines derived from double ditelosomic stocks of a hexaploid wheat cultivar were used to sequence each individual chromosome arm (except 3B) using Illumina short-reads technology (International Wheat Genome Sequencing Consortium (IWGSC), 2014). The chromosomes were assembled *de novo*, which reduced the complexity of assembling this highly redundant genome, aiding the differentiation of genes present in multiple copies and of highly conserved homologs.

A second approach involves sequencing a diploid progenitor species to aid in the assembly of the cultivated form. Care must be taken to choose the diploid progenitors most similar to the cultivated form. The diploid genomes of progenitor species can be used to determine the origin and structure of contigs when assembling large polyploid genomes. For example, strawberry (*Fragaria* × *ananassa*) is an octoploid (2n = 8x = 56) whose origin remains controversial. One theory suggests that it was formed from a natural hybridization between two octoploids- *F. virginiana* and *F. chiloensis* (Darrow, 1966). According to Davis et al. (2007), *F. vesca, F. nubicola*, and *F. orientalis* are possible progenitors. To access the genetic diversity of this valuable crop, one diploid variety of *F. vesca* (2n = 2x = 14) (*F. vesca* spp. *vesca* accession Hawaii 4) was sequenced (Shulaev et al., 2011).

Oilseed rape or canola (*B. napus)* is an allopolyploid derived from two diploid species of *Brassica* that are triplicated versions of an ancestral diploid. Genome assemblies of *B. napus* were assigned to these two subgenomes using sequence assemblies from each diploid progenitor, but many sequence scaffolds showed ambiguous assignment to homeologous groups, owing to homeolog exchange and frequent gene loss (Chalhoub et al., 2014). A similar strategy was used to characterize the allotetraploid genome of peanut (*Arachis hypogaea)*, which formed from two diploid species *A. duranensis* (A genome) and *A. ipaënsis* (B genome). Essentially complete assemblies of the genomes of the progenitor species *A. duranensis* and *A. ipaënsis* were generated and shown to directly align with the genetic map of a cultivated tetraploid peanut (Bertioli et al., 2015). In the same study, synthetic long-read sequencing of the tetraploid peanut genome showed that it was 98–99% identical to the diploid genomes, with differences due to recombination of polyploid genomes involved from the sequencing of DNA from purified chromosome arms (Bertioli et al., 2015). Some of the challenges in assembling the cultivated peanut genome have been the high similarity between the two-progenitor species, a high number of transposable elements, and recent evidence of tetrasomic recombination in this allotetraploid (Bertioli et al., 2015). Lastly, upland cotton (*G. hirsutum*) is an allotetraploid that formed 1–2 Myr (million years) ago from two unknown diploid progenitor species. The genome complexity of upland cotton was reduced by sequencing highly homozygous allohaploid lines to a coverage depth of 245x with Illumina short-read sequencing reads (Li et al., 2015). A dense genetic map was used to align and correct scaffolds, which covered 96% of the estimated 2.5 Gb genome, and fluorescence *in situ* hybridization (FISH) was used to confirm a successful allotetraploid assembly.

### Genome sequencing and algorithmic (pipeline) approach

There are several examples of successful *de novo* sequencing and assembly of large allopolyploid genomes of crops that use long-range alignments of sequence scaffolds to generate extended haplotypes to form distinctive homeologous pseudomolecules. Tobacco (*Nicotiana tabacum;* 2n = 4x = 48) is an allotetraploid that is derived from the diploid genomes of *N. sylvestris* and *N. tomentosiformis*. Whole-genome shotgun assemblies were aligned to physical maps to create longer super scaffolds that could be assigned directly to the progenitor genomes (Sierro et al., 2013). The polyploid genome of Indian mustard (*B. juncea*) (Yang et al., 2016) has been assembled using a combination of Illumina short reads, PacBio single molecule, real-time long sequence reads and optical maps from BioNano Genomics. The short and long reads were aligned to the maps, which directly helped in the determination of the individual molecules of tagged DNA, and dense genetic maps. The genome was almost fully represented in the assembly, which was assigned to the A genome [402 Megabase (Mb)] and the B genome (547 Mb).

Furthermore, an alternative approach to resolve polyploid complexity is by haplotyping. The process of assigning variants to a particular chromosome or defining which alleles appear together (corresponding haplotypes), is called phasing and haplotyping, respectively (Huang et al., 2017). Haplotypes can provide more information than un-phased genotypes in diverse fields, such as identifying genotype-phenotype associations and exploring genetic resistance to plant diseases. An example of this approach is the recent assembly of the hexaploid genome of sweetpotato (*Ipomoea batatas)*. The authors describe haplotype construction by applying a novel approach (Yang et al., 2017) where paired reads and mate pairs were initiatlly used for *de novo* assembly, then haplotypes were phased. Overlapping haplotypes were merged into larger haplotypes, mapping all the raw reads against the phased haplotypes. Finally, scaffolds were constructed based on the haplotypes and a consensus sequence was generated (Yang et al., 2017). This method, called “Ranbow,” can be downloaded at https://www.molgen.mpg.de/ranbow. A number of algorithms/tools to resolve the haplotype of polyploid genomes exist. Some examples are HANDS (Mithani et al., 2013), SDhaP (Das and Vikalo, 2015), and HapTree (Berger et al., 2014). Haplotype construction depends on the read depth or coverage as it is necessary to have a high coverage for each homolog (5–20x per homolog), as well as an insert size of 600–800 bp (Motazedi et al., 2017). It is also important to know the nature of the plant genome and ploidy before performing haplotyping in order to select the most appropriate tool. If available, it may be better to combine various individuals or parental information for haplotyping analysis (Motazedi et al., 2017). From an algorithmic point of view, haplotyping requires a lot of memory and computation time.

Another solution is the construction of a pan-genome, which shows the variation and commonality between individuals. A pan-genome includes “completeness” as it contains the core genome shared by all the individuals sequenced, but also the genes that are absent/present in some of the re-sequenced genomes. Generally, it is a very helpful approach for breeding applications as it anchors all the known variations and phenotype information and can include wild relatives of the cultivated crop lines. It also aids in the identification of novel genes from the available germplasm that are not found in the reference genome (The Computational Pan-Genomics Consortium, 2016). Additionally, it represents the polyploid genomes and in the case of the allopolyploids, it allows the quantification of allele dosage between germplasm samples (The Computational Pan-Genomics Consortium, 2016). Pan-genome construction is even more computationally challenging in the case of polyploid plant genomes as the corresponding genotype needs to be determined by variant calling and identifying novel variants for all the haploids. Previously, a pan-genome was constructed from 18 wheat cultivars and it was shown that a large number of variable genes affected by presence/absence and variation between the genes could be associated with important agronomic traits (Montenegro et al., 2017). NRGene's (www.nrgene.com) PanMAGIC platform can be used for pangenome analysis and was applied to analyze six maize genomes (Lu et al., 2015).

## Third generation genomic technologies come to the rescue

Genome assembly and scaffolding can be performed using shorter reads (Illumina data), or longer reads from either PacBio (www.pacb.com) or Oxford Nanapore (https://nanoporetech.com/), or a combination of both short and long reads. Another alternative is the assembly of linked reads from 10X genomics. Additionally, for higher contiguity, longer-range scaffolders from Dovetail (dovetailgenomics.com) and BioNano Genomics (bionanogenomics.com) can be used for the construction of physical maps using very large DNA fragments. A hybrid scaffolding approach can also be applied where longer reads are used to improve assemblies generated using short-reads or even combined wiht longer-range scaffolding data.

Even though the hexaploid wheat genome was assembled from only short reads, it is very challenging to assemble such a large and highly repetitive genome using this approach. A less complicated assembly strategy is to use long-reads to aid in the assembly of difficult portions of the genome. The most widely used long-read sequencing technology is Pacific Biosciences' Single Molecule Real-Time (SMRT) sequencing. Recently, a few polyploid plant genomes were assembled using PacBio long reads including three allotetraploid plant genomes *C. quinoa* (quinoa) (Jarvis et al., 2017), *Eleusine coracana* (finger millet) (Hatakeyama et al., 2017) and *Coffea arabica* (Arabica coffee) (Cheng et al., 2017).

As mentioned earlier, another solution to the read length issue is the ultra-long and real-time data sequencing approach by Oxford Nanopore Technologies (www.nanoporetech.com). Currently three plant genomes have been sequenced with Nanopore, a wild tomato genome *Solanum pennellii* (Schmidt et al., 2017), the genome of *A. thaliana* (Mondal et al., 2017), and most recently the genome of *Oryza coarctata* (Michael et al., 2018). Illumina's SLR technology on the other hand, has already been applied for the estimation of the haploid draft genome of the polyploid sugarcane hybrid SP80-3280 (Riaño-Pachón and Mattiello, 2017).

The long-reads can also be combined with existing short-reads for genome assembly, called hybrid genome assembly. The resulting genome assembly from short-reads needs improvement in its contiguity because the contigs need to be assembled into scaffolds. Initially, the contigs are ordered using alignments from paired-end reads, read pairs from (Bacterial Artificial Chromosome) BAC or fosmid ends, which are powerful ways to increase the contiguity and help bridge the repeats—the main reason generally for breaks in the genome assemblies. In addition, genetic and physical maps are also essential for polyploid plant genome assembly (i.e., a physical map was used in the case of the tetraploid cotton genome). Optical mapping enables the fingerprinting of large genome fragments and can be used to improve highly fragmented genome assemblies. This technology promises the improvement of scaffolding and eventually lessens the need for genetic and physical mapping (Jiao and Schneeberger, 2017).

Another new promising technology that can potentially be applied to complex, polyploid plant genomes is the 10X genomics approach. There is only one scientific report on plant research using this technology to date on a diploid pepper genome (*Capsicum annuum*) (Hulse-Kemp et al., 2018). The haplotype construction was generated to karyotype aneuploidy in a cancer study (Bell et al., 2017) and it was also used in the generation of a protocol for haplotyping human genome (Porubsky et al., 2017), making it a promising technique for polyploidy genome data. Additional techniques used by polyploid plant projects include Hi-C and chromosome-scale assembly. For example, a study is underway to detect large chromosomal rearrangements in wheat genomes (Monat et al., 2018) and another project uses chromosome scale scaffolding on the allotetraploid coffee genome (Zimin et al., 2018).

## Advances in genomic resources and functional tools in molecular genetics and breeding

The advance of NGS technologies has immensely impacted the field of plant genomics in model and non-model crops alike, and it is continuously contributing to bridging the gap between genotype and phenotype. The genotype can be linked to the phenotype by Genome Wide Association studies (GWAS) and the advent of NGS has revolutionized genomic, as well as, transcriptomic (RNA-Sequencing) approaches to biology including plant genomics in model and non-model crops. Modern breeding programs combine various approaches for more efficient breeding, in parallel with the reduction of the whole breeding period (Varshney et al., 2013). These approaches include the traditional phenotype-based selection, marker-assisted selection, and genome-assisted breeding (Varshney et al., 2013). The continuous effort in improving major crops has resulted in great genetic and genomic resources for crop traits. Some instances of databases that host these resources can be found in Table 3.

**Table 3:** Host-databases of various plant genetic and genomic resources.

**DB name**	**Resources**	**Plants**	**URL**
Genbank	Genomic	Various plant species	https://www.ncbi.nlm.nih.gov/genbank/
EMBL	Genomic	Various plant species	https://www.ebi.ac.uk/
DDBJ	Genomic	Various plant species	http://www.ddbj.nig.ac.jp/
UniProt	Protein and functional	Various plant species	http://www.uniprot.org/
NCBI	Genomic	Various plant species	https://www.ncbi.nlm.nih.gov/
GOLD	Genomic, metagenomics, transcriptomic	Various plant species	https://gold.jgi.doe.gov/cgi-bin/GOLD/bin/gold.cgi
Phytozome	Genomic	92 assembled and annotated plant species	https://phytozome.jgi.doe.gov/pz/portal.html
Plantgdb	Genomic, transcriptomic	27 assembled and annotated plant species	http://www.plantgdb.org/
Sol	Genomic	11 *Solanaceae* species	https://solgenomics.net/
Gramene	Genomic, genetic markers, QTLs	53 plant species	http://www.gramene.org/
MaizeGCB	Genomic, annotations, tool host	*Zea mays*	https://www.maizegdb.org/
Tair	Genetic and molecular biology data	*Arabidopsis thaliana*	https://www.arabidopsis.org/
CottonGEN	Genomic, Genetic and breeding resources	49 *Gossypium* species	https://www.arabidopsis.org/
PLEXdb	Gene expression	14 plant species	http://www.plexdb.org/
RicePro	Gene expression	*Oryza sativa*	http://ricexpro.dna.affrc.go.jp/
CerealsDB	Genetic markers	*Triticum aestivum*	http://www.cerealsdb.uk.net/cerealgenomics/CerealsDB/indexNEW.php
PeanutBase	Genome, MAS, QTLs, Germplasm	*Arachis hypogaea*	https://peanutbase.org/
SoyKb	Genetic markers, genomic resources	*Glycine max*	http://soykb.org/
SoyBase	Genetic markers, QTLs, genomic resources	*G. max*	https://soybase.org/
PGDBj	Genetic markers, QTLs, genomic resources	80 plant species	http://pgdbj.jp/
SNP-Seek	Genotype, Phenotype and Variety information	*O. sativa*	http://snp-seek.irri.org/
GrainGenes	Genome, Genetic markers, QTLs, genomic resources	*T. aestivum, Hordeum vulgare, Secale cereale, Avena sativa* etc	https://wheat.pw.usda.gov/GG3/
ASRP	small RNA	*A. thaliana*	http://asrp.danforthcenter.org/
CSRDB	small RNA	*Z. mays*	http://sundarlab.ucdavis.edu/smrnas/
BrassicaInfo	Genomic	7 *Brassica* species	http://brassica.info/
BRAD	Genomics, Genetic Markers and Maps	*Brassica*	http://brassicadb.org/brad/
Ensembl Plants	Genomic	45 plant species	http://plants.ensembl.org/index.html
Ipomoea Genome Hub	Genomic, EST	*Ipomoea batatas*	https://ipomoea-genome.org/
PGSC	Genomic, annotation	*S. tuberosum, S.chacoense*	http://solanaceae.plantbiology.msu.edu/pgsc_download.shtml
GDR	Genomics, Genetics, breeding	*Rosaceae*	https://www.rosaceae.org/analysis/266
HWG	Genomics, Transcriptomics, Genetic Markers	Forest trees and woody plants	https://www.hardwoodgenomics.org/

## Lack of complexity of the currently available reference genomes of polyploid crops

High quality reference genomes, gene discovery, and comparative genomics depend on the construction of a high quality *de novo* genome assembly. These assemblies are more feasible, but still not perfect using haploid and inbred species. Despite their importance to reflect the genetic information within an organism, most of the currently available polyploid and diploid plant genome assemblies do not capture the heterozygosity present. The majority of the currently available reference genomes, especially those of the polyploids, lack variation and characteristics of other individuals that are not captured or presented. This happens because the simpler genomes are sequenced first, but also due to the sequencing of diploid and less heterozygous progenitor species for the reduction of the intricacy of the polyploid assembly problem. In reality, the assembled genome is a flat DNA sequence, which shows neither the variation between homologous chromosomes, nor allelic variations, or structural variations. The resulting “model” reference genome is more distant than the majority of the other individuals in a species. Furthermore, genes may be missing or not annotated. A solution to this problem is the construction of pan-genomes (as described above), which show the core and the variable regions of a genome between individuals. An example of a pan-genome application is in the hexaploid bread wheat (Montenegro et al., 2017).

Even in the case of the smaller, “simpler” bacterial genomes, the submitted genomes are not complete. Despite the exponential generation of NGS data, the majority of the submitted genomes represent only draft or in scaffold format, incomplete genomes. The higher ploidy levels of the polyploid plant genomes make the situation even more difficult to handle. This leads to highly fragmented genome assemblies, with disconnected contigs of repetitive sequences. As discussed, better tools are needed that allow automatic contig assembly of (plant) genomes with many repeats and that are sensitive to ploidy levels and can handle haplotype construction. Also, to date allopolyploid plant genomes cannot be represented in an integrated assembly, rather the sub-genomes are found in separate assemblies.

## Conclusions

Improving genome sequencing and assembly of polyploid plant crops will have a fundamental impact on genetic research and on plant breeding by better understanding the genomes, identifying genomic variants and relating them to economic, physiological, and morphological agronomic traits, such as higher yield, abiotic/biotic tolerance, root structure etc. Better polyploid plant genome assemblies will also aid in the study of the genotype-phenotype-environment relationship. For this, more plant polyploid-oriented algorithmic and technological (sequencing) advances are necessary. High quality reference sub-genomes in polyploid crops in addition to multiple reference genomes or a pan-genome per crop species are necessary to capture variation and to better understand these economically important genomes.

## Author contributions

MK: drafted the manuscript, compiled the tables, and made the figure. MK, NA, DE, HT, and MS: designed the outline, content, and edited the manuscript.

### Conflict of interest statement

The authors declare that the research was conducted in the absence of any commercial or financial relationships that could be construed as a potential conflict of interest.
